# Determinants of perceived patient benefit in a longitudinal cohort study of patients with psoriasis and atopic dermatitis

**DOI:** 10.1038/s41598-024-84794-2

**Published:** 2025-01-10

**Authors:** Gloria-Beatrice Wintermann, Susanne Abraham, Eva M. J. Peters, Stefan Beissert, Kerstin Weidner

**Affiliations:** 1 Department of Psychotherapy and Psychosomatic Medicine, Faculty of Medicine, Technische Universität Dresden, Dresden, Germany; 2 Department of Dermatology, University Hospital Carl Gustav Carus Dresden, Technische Universität Dresden, Dresden, Germany; 3 Psychoneuroimmunology Laboratory, Department of Psychosomatic Medicine and Psychotherapy, Justus-Liebig University Giessen, Giessen, Germany; 4 Department of Psychosomatic Medicine and Psychotherapy, CharitéCenter 12 Internal Medicine and Dermatology, Charité-Universitätsmedizin Berlin, Berlin, Germany

**Keywords:** Psoriasis (PSO), Atopic dermatitis (AD), Patient benefit index (PBI), Dermatology life quality index (DLQI), Anxiety/depression, Biologics, Systemic therapy, Psychology, Diseases, Health care, Medical research, Risk factors, Signs and symptoms

## Abstract

The Dermatology Life Quality Index (DLQI) should be used to assess treatment success in psoriasis (PSO). However, the DLQI does not assess the importance and achievement of treatment goals. The Patient Benefit Index (PBI) is a questionnaire that takes both into account. Currently, there is insufficient knowledge about the modulating variables of the PBI and whether it can complement the assessment of the DLQI. In a longitudinal cohort study, 82 patients with PSO were assessed before and up to sixteen weeks after a new treatment episode. The PBI was compared with patients with atopic dermatitis (AD) (*n* = 61). The effects of gender, age, type of therapy, improvement in body surface area (BSA), anxiety/depression, DLQI and individual coping were assessed. “Getting better skin quickly” was most important in PSO. Improved BSA, anxiety/depression, DLQI, male gender and initiation of biological therapy had the most positive effects. Partial mediation was found for the reduction of anxiety/depression and improved coping. The PBI may be considered an appropriate outcome measure of treatment success in PSO, complementing the DLQI. Patients with clinically relevant anxiety/depression and inadequate coping should be offered adjuvant psychosomatic treatment.

**Supplementary Information:**

The online version contains supplementary material available at 10.1038/s41598-024-84794-2.

## Introduction

Psoriasis (PSO) is a chronic inflammatory dermatological, systemic and immune-mediated disease affecting ~ 2% of the Western or Central European population. Incidence rate is high, e.g. ~ 521 per 100 000 person-years in Western Europe - with higher values in adults, high-income countries, older populations^1^. It is characterised by red, scaly plaques and psoriatic lesions, most commonly on the elbows, knees, scalp and lower back. Metabolic, arthritic and cardiovascular comorbidities are common, as well as psychological disorders - with a 1.5 times higher risk of depressive symptoms and an even higher prevalence of anxiety symptoms (20–50%) compared with non-affected patients^2^. Due to pain, pruritus and feelings of stigmatisation, patients with PSO suffer a tremendously reduced quality of life^3,4^. As a result, the World Health Organisation has recognised PSO as a serious non-communicable disease World Health^5^.

Systemic agents with interleukin inhibitors have been shown to be effective in treating moderate to severe PSO^6^. Data show higher patient-reported therapeutic benefit with biologics than with conventional systemic treatments^7^. There is currently a lack of knowledge about the variables that influence treatment success, as measured by patient-reported satisfaction with treatment outcomes.

The S3 guideline for the treatment of PSO recommends the Dermatology Life Quality Index (DLQI) as a patient-reported outcome measure in addition to dermatologist assessment of the extent and severity of psoriatic skin^8^. The DLQI measures the severity of PSO by considering the impact of PSO on feelings and daily life (e.g. work, leisure activities, personal relationships)^9^. However, it has some shortcomings, for example in terms of construct validity. It is supposed that the DLQI does not capture the true severity of PSO, as patients can answer ‘not relevant’ to several items. Above, some items assess more than one aspect at the same time (e.g. inference with going shopping or looking after your home). Both can lead to an underestimation of the severity of the disease and therefore the perceived success of treatment^9–11^. There is evidence that patients with higher objective disease severity are particularly likely to rate items as ‘not relevant’^12,13^. As a result, the findings suggest a low correlation between disease severity and impact on health-related quality of life^14–17^. In addition, the DLQI does not take into account the importance of patients’ individual treatment goals, which would help to better tailor dermatological treatment and understand the reported treatment benefit. Taking the patient’s needs into account would lead to greater treatment satisfaction and adherence^18^. The Patient Benefit Index PBI^19,20^, is a patient-reported outcome measure that assesses both, the importance of treatment goals using the Patients Needs Questionnaire (PNQ), and the perceived benefit of treatment, using the Patients Benefit Questionnaire (PBQ). At present, it is not clear whether the PBI can adequately complement the DLQI and contribute to a more accurate measurement of self-reported treatment success. Above, there is a lack of evidence on the modulating variables of the PBI in PSO. Previous studies have shown that individual treatment goals vary with different influencing variables^18,21^. In addition to clinical characteristics, e.g. skin disease severity, sociodemographic variables such as age and gender may also have an impact^21^. Another study showed that the perceived benefit of therapy decreases as the number of prior systemic treatments increases. It was lower for prior conventional systemic - and lowest for prior biologic therapies^21^. Another study showed correlations with the DLQI^22^. Above, it is known from a large European multi-centre study that clinically relevant depression (~ 14%) and anxiety (~ 23%) are common in PSO^23^. This suggests that in addition to sociodemographic and clinical variables, patient-reported outcomes such as quality of life and anxiety/depression may also influence perceived treatment benefit. This may be clinically relevant in order to provide appropriate, targeted dermatological treatments and adjunctive psychosomatic interventions to specific patient subgroups.

At present, longitudinal studies investigating the influence of clinical, sociodemographic and psychological variables on perceived treatment benefit in patients with PSO are lacking. Differences in the importance of treatment goals and perceived treatment benefits between dermatological subgroups and the mediating role of psychological variables have not yet been investigated. Thus, the following study investigated the needs and benefits of patients with PSO and compared them with a dermatological control group of patients with atopic dermatitis (AD). Above, the influence of clinical, sociodemographic and psychological variables on the PBI was examined. Finally, we were interested in the possible mediating role of anxiety/depression and coping strategies.

## Methods

### Design

Patients with a primary dermatological diagnosis of psoriasis (PSO) or atopic dermatitis (AD) were consecutively enrolled in this observational, single-centre, prospective cohort study between October 2015 and September 2019. The present study was part of a research topic, called “The assessment of psychological predictors of the therapy outcome in patients with psoriasis”^24^. The study was not registered on any registration platform.

### Study sample

Inclusion criteria were as follows: male and female patients with PSO or AD; between 18 and 75 years of age; at least moderate severity of the skin disease (as assessed by a dermatologist); initiation of a new treatment episode (e.g. change from systemic therapy to biologic therapy or from one systemic therapy to another in PSO or initiation of intensified local therapy in PSO and/or AD); adequate knowledge of German; complete data for the primary outcome measures (Body Surface Area/BSA; PBI; DLQI; Hospital Anxiety and Depression Scale/HADS). Exclusion criteria were: refusal to participate, inability to give informed consent (e.g. due to mental retardation, dementia, acute psychosis or language barriers); a sufficiently long washout period (at least 12 weeks); an exclusive diagnosis of psoriasis intertriginosa.

### Procedure

The majority of patients (PSO: *n* = 56, 68.3%; AD: *n* = 46, 75.4%) were enrolled in an inpatient unit belonging to the Department of Dermatology at a large university hospital in Germany. In addition, recruitment took place in two outpatient clinics, one specialising in the diagnosis and treatment of PSO and one specialising in AD, both located in the same department. At the first time point (T1), before the start of a new treatment episode, e.g. in the form of intensified local therapy with/without conventional systemic or biological treatment, patients were asked to complete several questionnaires. The extent and severity of the psoriatic lesions or eczema were assessed by a dermatologist (under the supervision of the co-author SA) to determine at least moderate severity of the skin disease. Approximately twelve to 16 weeks after successful enrolment (T2), patients were asked to complete the same questionnaires again. In addition, the severity of the skin condition was again assessed by a dermatologist. However, as dermatological treatment was continued in outpatient clinics outside the university hospital where recruitment took place, an objective measure of skin disease severity was not available for most patients (for PSO: *n* = 54/65.9% missing values, for AD: *n* = 44/72.1% missing values).

### Measures

#### Patient-reported outcomes (PRO)

The following PRO measures and questionnaires were used both before (T1) and after (T2) the start of a new treatment episode: The Patient Benefit Index (PBI)^20^ was used as the primary outcome measure. It consists of the Patient Need Questionnaire (PNQ) administered at T1 and the Patient Benefit Questionnaire (PBQ) administered at T2. The 25 treatment goals of the PNQ are rated on a 5-point Likert scale according to their importance. Similarly, the PBQ assesses the extent to which treatment goals were achieved. The PBI is calculated by taking the score for each item on the PNQ and multiplying it by the corresponding item on the PBQ. Each product is divided by the sum of all individual importance scores. Finally, the sum of each product is calculated and corresponds to the PBI (range 0–4, no benefit to maximum benefit) (Cronbach’s α PNQ PSO/AD, T1 = 0.88/0.87, PBQ, T2 = 0.96/0.96). The higher the PBI, the better the fit between patient needs and benefits. A PBI of ≥ 1 can be considered as the minimum patient-relevant benefit.

The *Dermatology Life Quality Index* (DLQI)^25^ assesses the impact of the skin condition on daily life over the past seven days. The 10 items are scored on a 4-point scale (range 0–30) (Cronbach’s alpha PSO/AD, T1, T2 = 0.90/0.89, 0.92/0.91). The higher the DLQI score, the lower the skin-related quality of life.

The *Hospital Anxiety and Depression Scale* (HADS)^26,27^ is a 14-item self-report measure used to assess symptoms of anxiety and depression in somatically ill patients during the past week, rated on a 4-point Likert scale (range 0–21) (Cronbach’s alpha PSO/AD, T1/T2 = 0.89/0.84, AD: 0.91/0.88).

The *Marburg Skin Questionnaire (Marburger Hautfragebogen) (MSQ) for for coping with skin diseases*^28^ assesses the degree of coping with psoriasis/AD, using six subscales (social anxiety, itch-scratch cycle, helplessness, depression/anxiety, quality of life, information seeking, ), at T1 and T2. Items are rated on a 5-point Likert scale (1 = not at all to 5 = very strongly) and summed to a subscale score. Higher scores correspond to a more negative, lower scores to a more positive assessment (Cronbach’s alpha PSO/AD, T1 = 0.95/0.77 social anxiety; 0.78/0.87 itch-scratch cycle; 0.83/0.87 helplessness, 0.89/0.82 depression/anxiety,. 77/0.65 quality of life, 0.78/0.71 information seeking; T2 = 0.95/0.96 social anxiety, 0.93/0.91 itch-scratch cycle, 0.89/0.90 helplessness, 0.75/0.87 depression/anxiety, 0.80/0.80 quality of life, 0.78/0.71 information seeking).

The *Subjective Psoriasis Area and Severity Index* SAPASI^29^, was used as a self-reported measure of the psoriasis severity. The formula according to Feldman et al.^30^ was used (range 0–72). Accordingly, a score between 3 and 15 was considered moderate. Previous studies have shown a high correlation between SAPASI and PASI^30,31^.

The *Body Surface Area* (BSA) is a measure of the proportion (%) of the skin area affected by PSO or AD. Patients draw in the areas covered by psoriatic lesions on a silhouette of the front and back of their body. A dermatologist (under the supervision of the co-author SA) then calculates the BSA. The area of an adult’s hand was used as a marker. A BSA of ≤ 10 can be considered as mild, > 10% as moderate to severe^32^. The BSA was used as the primary outcome to measure the severity of PSO, as complete data were available at both at T1 and T2. Existing findings could show a high correlation with the PASI^33^.

The *subjective SCORAD* (Scoring Atopic Dermatitis, European Task Force on Atopic Dermatitis^34^, was used in order to assess the severity and extent of eczema in patients with AD. The affected area (A) is calculated using the rule of nine, based on a drawing in which the extent of the eczema is marked by the patient. In addition, a representative area (B) of eczema is scored on the six dimensions (e.g. redness, swelling, oozing/crusting), using a four-point rating scale (0 = none, 1 = mild, 2 = moderate, 3 = severe). Subjective symptoms (C) of itching and sleeplessness are rated by the patient using a visual analogue scale ranging from 0 (no itch) to 10 (worst). The SCORAD index formula A/5 + 7B/2 + CA was used, resulting in a maximum score of 103. A score between 25 and 50 corresponds to a moderate severity of AD^35^.

#### Dermatologist-assessed measures

To calculate the *objective SOCRAD*, the extent of the affected skin area was assessed by a dermatologist (under the supervision of the co-author SA). A score ranging between 25 and 50 corresponds to a moderate severity of AD^35^. The *Psoriasis Area and Severity Index* (PASI)^9,36^ assesses the severity of PSO, including the criteria erythema, induration and desquamation on a 5-point scale, and the area of skin affected (for head, arms, legs, trunk) (maximum score 72). A score above 10 can be considered as moderate to severe severity^8^. In the present study, BSA was used as the primary outcome measure because PASI scores could only be obtained in 34.1% of the PSO patients, at both time points.

Structured questionnaires were used to collect additional clinical (e.g. onset of the skin disease, medications, former dermatological treatments/systemic or biologic therapies, somatic comorbidities, habitual smoking/alcohol consumption, Body Mass Index/BMI) and sociodemographic information (e.g. partnership, education, nationality). The diagnosis of a PSO or AD, additional somatic comorbidities, medications and type of new dermatological therapy were ascertained by reviewing the physician´s letter from the medical record.

#### Statistical analysis

We only analysed complete data for BSA, PBI, HADS and DLQI at both T1 and T2. Patients with missing data at T2 were defined as drop-outs (see Table S1 in the supplementary material). The drop-out analysis was calculated only for patients with PSO, as these were the main focus of the present study. For categorical variables, the number and percentage of cases were calculated. Normal distribution was checked using the Kolmogorov-Smirnov test. Continuous and normally distributed data were presented as means and standard deviations, ordinal or non-normally distributed data as medians and interquartile ranges. Group differences were tested using the Mann-Whitney U test. Categorical or dichotomous variables were analysed using chi-squared or Fisher’s exact test. To detect changes due to dermatological treatment, ordinal or non-normally distributed data were analysed using the Wilcoxon signed-rank test or, in the case of dichotomous variables, the McNemar test. The effect of the influencing variables on the PBI was calculated using univariable/multivariable regression analyses. Influencing variables included: change (delta/Δ) in skin severity, HADS, DLQI and coping, type of treatment option received, age and sex.

Mediator analyses were conducted to determine whether the potential effect of the treatment strategy on perceived patient benefit (PBI) was mediated by a reduction in anxiety/depression (ΔHADS). Above, it was of interest whether the possible effect of skin improvement (ΔBSA) on PBI is mediated by an improvement in coping with skin disease (ΔMSQ). The Process macro model, version 4.0 by Hayes et al.^37,38^ (https://www.processmacro.org/download.html) was used with bootstrapping (*n* = 5000 bootstrap samples) and heteroscedasticity-consistent inference. P-values were adjusted for multiple testing when appropriate (Bonferroni-Holmes correction). Otherwise, results were considered significant at a significance level of *p* ≤ .05.

## Sample size and power calculation

An a priori sample size calculation indicated that 85 patients were needed, to achieve a power of 80% (using multiple linear regression, four predictors and a medium effect size f^2^ = 0.14). Furthermore, assuming a drop-out rate of ~ 25%, according to Scharloo, et al.^39^, a total sample of 110 patients with PSO would have been required. An a posteriori power calculation (assuming a sample size of 82 patients with PSO with the same parameters) resulted in a power of 78.6% ^40^.

## Results

### Sociodemographic and clinical characteristics of the patient groups

82 patients with psoriasis (PSO) with a median age of 54.6 years, could be included in the final analyses. 70 patients (85.4%) were diagnosed with psoriasis vulgaris, *n* = 4 (4.9%) with psoriasis vulgaris et palmoplantaris, *n* = 6 (7.3%) with psoriasis palmoplantaris/plantaris, two patients with psoriasis guttata (2.4%). 48 patients (58.5%) received a new treatment episode with a combination of intensified local therapy and biologic therapy (*n* = 26 secukinumab, others: certolizumab, brodalumab, etanerecept, guselkumab, ustekinumab, tildrakizumab). 24 patients (29.3%) received a new treatment episode with a combination of intensified local therapy and conventional systemic therapy (e.g. apremilast, ciclosporin, fumaderm, methotrexate), ten patients (12.2%) received intensified local therapy with or without phototherapy. Of the patients with AD, *n* = 43 (70.5%) received an intensified local therapy or systemic therapy (e.g. cyclosporine, azathioprine) (combined with intensified local therapy) and *n* = 18 (29.5%) received a biologic (dupilumab).

Compared to the dermatological control group of patients with AD, the patients with PSO were older, more likely to be in a partnership and had a higher BMI (see Table 1). Patients with PSO were more likely to have circulatory and liver diseases, whereas patients with AD were more likely to have allergies, autoimmune and pulmonary diseases as somatic comorbidities (see Table S1).

**Table 1:** Sociodemographic and life-style characteristics of patients with psoriasis (PSO) (*n* = 82) and patients with atopic dermatitis (AD) (*n* = 61) before (T1) the beginning of a new treatment episode.

Patients´ characteristics	Patients with PSO (*n* = 82)	Patients with Atopic Dermatitis (AD) (*n* = 61)	U/Z/T/χ^2^ (*p*)
Age, median (IQR), min-max Range	54.6 (38.2–62.6), 22.9–74.9	43.8 (29.5–59.8), 19.8–74.2	1960.000 (U) (0.027*)
Gender, n (%)			
Women	29 (35.4)	27 (44.3)	1.162 (0.281) (χ^2^)
Men	53 (64.6)	34 (55.7)	
Education, n (%)			
No school degree/Less than 10 years	12 (14.6)	8 (13.1)	2.664 (0.264) (χ^2^)
10 years	51 (62.2)	30 (49.2)	
Abitur	19 (23.2)	21 (34.4)^1^	
Partnership, n (%)			
Married	36 (43.9)	24 (39.3)	5.693 (0.058) (χ^2^)
Cohabited	31 (37.8)	15 (24.6)	
No partnership	15 (18.3)	21 (34.4)^2^	
Regular sport, n (%)			
Yes	23 (28.0)	21 (34.4)	2.135 (0.344) (χ^2^)
No	59 (72.0)	39 (63.9)^3^	
Regular alcohol, n (%)			
Yes	39 (47.6)	33 (54.1)	
No	43 (52.4)	28 (45.9)	0.598 (0.439) (χ^2^)
Smoking, n (%)			
Yes	21 (25.6)	13 (21.3)	0.361 (0.548) (χ^2^)
No	57 (69.5)^1^	45 (73.8) 4	
BMI kg/m^2^,			
Median (IQR), min-max Range	27.1 (24.6–31.6), 17.0-53.9	24.9 (4.9), 17.0–43.0	1535.000 (< 0.001***) (U)
		23.6 (21.8–26.7)	

*Psoriasis*, missing values:^1^ *n* = 4; *Atopic dermatitis*, missing values:^1^ *n* = 2^2^, *n* = 1^3^, *n* = 1^4^, *n* = 3; *p ≤ .05, ** *p* ≤ .01, ***p ≤ .001.

Above, patients with PSO had a lower body surface area affected by the skin lesions, typical of the respective chronic inflammatory skin disease, measured before the onset of a new treatment episode, compared to patients with AD (U = 1477.000, *p* < .001). Both groups showed a significant reduction in symptom severity when BSA and the subjective symptom severity scores (SAPASI, subjective SCORAD) were considered (*p* < .001). In addition, the skin-related quality of life improved significantly in both groups (*p* < .001), although the latter was worse in patients with AD than in PSO, at both time points (T1: U = 1756.500, *p* = .002; T2: U = 1551.500, *p* < .001). At the same time, patients with AD had significantly higher HADS anxiety/depression scores, only at T1 (U = 1861.500, *p* = .020), which improved significantly over time (Z = -2.292, *p* = .022), whereas the score remained stable in patients with PSO (Z = − 0.386, *p* = .699) (see Table 2).

Patients with PSO who were successfully enrolled in the present study were less likely to be smokers, had a higher health-related quality of life, and were less anxious/depressed compared to patients who dropped out (see Table S3 in supplementary material).

### Patient needs and perceived patient benefit index (PBI)

More than three quarters of patients with PSO (84.1%) and AD (77.0%) achieved more than minimal benefit (PBI ≥ 1) from the new treatment episode. Above, the two groups did not differ significantly in terms of PBI (F(1,143) = 3.671, *p* = .057). The three most important needs were ‘to get better skin quickly’ (84.1%), ‘to be cured of all skin defects’ (81.7%) and ‘﻿to find a clear diagnosis and therapy﻿’ (78.0%) for patients with PSO. For patients with AD, the most important treatment goals were ‘to be free of itching’ (90.2%), ‘to get better skin quickly’ (82.0%) and ‘no longer have burning sensations on your skin’ (75.4%) were (see Table 2). For patients with AD, the treatment goals ‘to be free of itching’ and ‘to be able to sleep better’ were significantly more important than for patients with PSO (p ≤ .05 after Bonferroni-Holm correction) (Table 2).

**Table 2:** Patient needs and perceived patient benefit (PBI = patient benefit index) as compared between patients with psoriasis (PSO) (*n* = 82) and patients with atopic dermatitis (AD) (*n* = 61).

Patients´ characteristics	Patients with PSO (*n* = 82)	Patients with AD (*n* = 61)	F/U/χ^2^ (*p*)
Patient Benefit Index, mean (SD), min-max Range	2.3 (1.1), 0.0–4.0	1.9 (1.1), 0.0–4.0	3.671 (0.057) (F)^a^
PBI ≥ 1 (minimum patient-relevant benefit), n (%)	69 (84.1)	48 (78.7)	0.700 (0.403) (χ^2^)
PNQ-Items, mean (SD)^1^, n (%) answering “very important”			
Patient Need 1: be free of pain	2.7 (1.7), 48 (58.5)	2.4 (1.7), 25 (41.0)	2151.500 (0.121)
Patient Need 2: be free of itching	3.4 (1.2), 57 (69.5)	3.8 (0.6),^2^ 55 (90.2)	1906.500 (0.001***)^b^
Patient Need 3: no longer have burning sensations on your skin	2.9 (1.7), 53 (64.6)	3.6 (0.9), 46 (75.4)	2113.500 (0.052)
Patient Need 4: be cured of all skin defects	3.7 (0.7), 67 (81.7)	3.5 (1.0), 46 (74.2)	2322.000 (0.303)
Patients Need 5: be able to sleep better	2.0 (1.8), 28 (34.1)	3.0 (1.3), 30 (49.2)	1784.500 (0.002**)^c^
Patients Need 6: feel less depressed	2.0 (1.7), 24 (29.3)^2^	2.6 (1.5), 26 (42.6)	1955.000 (0.028*)
Patients Need 7: experience a greater enjoyment of life	2.5 (1.6), 35 (42.7)^2^	2.9 (1.4), 30 (49.2)	2200.500 (0.238)
Patients Need 8: have no fear the disease will become worse	3.1 (1.3), 44 (53.7)	2.9 (1.3), 28 (45.9)	2288.500 (0.349)
Patients Need 9: be able to lead a normal everyday life	3.0 (1.5), 45 (54.9)	3.3 (1.1), 39 (63.9)	2205.500 (0.174)
Patients Need 10: be more productive in everyday life	2.3 (1.6), 29 (35.4)	2.9 (1.3)^1^, 27 (44.3)	2053.500 (0.080)
Patients Need 11: be less of a burden for relatives and friends	2.3 (1.6), 25 (30.5)^2^	2.8 (1.5), 31 (50.8)	1962.500 (0.029*)
Patients Need 12: be able to engage in normal leisure activities	2.9 (1.4), 41 (50.0)	2.8 (1.4), 29 (47.5)	2384.500 (0.610)
Patients Need 13: be able to lead a normal working life^1^	1.9 (1.8), 31 (37.8)	2.6 (1.7), 29 (47.5)	2055.000 (0.068)
Patients Need 14: be able to have more contact with other people	2.1 (1.6), 20 (24.4)^2^	2.3 (1.5), 19 (31.1)	2237.000 (0.323)
Patients Need 15: be comfortable showing yourself more in public	2.4 (1.5), 29 (35.4)	2.4 (1.5), 19 (31.1)	2421.500 (0.736)
Patients Need 16: be less burdened in your partnership	2.4 (1.7), 31 (37.8)^2^	2.4 (1.7), 24 (39.3)	2431.500 (0.866)
Patients Need 17: be able to have a normal sex life	2.3 (1.7), 30 (36.6)^2^	2.5 (1.5), 21 (34.4)	2385.000 (0.714)
Patient Need 18: be less dependent on doctor and clinic visits	3.5 (0.8), 53 (64.6)^2^	3.5 (0.9), 41 (67.2)	2436.500 (0.867)
Patients Need 19: need less time for daily treatment	3.3 (1.1), 48 (58.5)	3.1 (1.0), 28 (45.9)	2193.500 (0.168)
Patients Need 20: have fewer out-of-pocket expenses	2.9 (1.4), 40 (48.8)^2^	2.8 (1.3), 26 (42.6)	2363.500 (0.639)
Patients Need 21: have fewer side effects	3.0 (1.4), 48 (58.5)	2.8 (1.4), 30 (49.2)	2269.000 (0.299)
Patients Need 22: find a clear diagnosis and therapy	3.6 (1.0), 64 (78.0)	3.5 (0.9), 41 (67.2)^2^	2241.000 (0.240)
Patients Need 23: have confidence in the therapy	3.6 (0.9), 59 (72.0)	3.5 (0.9), 40 (65.6)^3^	2299.500 (0.534)
Patients Need 24: get better skin quickly	3.7 (0.8), 69 (84.1)	3.7 (0.7), 50 (82.0)	2445.000 (0.725)
Patient Need 25: regain control of the disease	3.5 (1.0), 60 (73.2)	3.5 (0.9), 41 (67.2)	2381.500 (0.541)

^1^ Means and standard deviations are shown instead of medians and interquartile ranges (if there is no normal distribution), in order to be able to see group differences more easily.

^2^ one missing value^3^, two missing values; ^a^ controlled for age; ^b^*p* = .03 after Bonferroni-Holm correction, ^c^*p* = .05 after Bonferroni-Holm correction; *p ≤ .05, ** *p* ≤ .01, ***p ≤ .001.

### Influencing variables on PBI

Female patients with PSO had a lower PBI than male patients (Beta =-0.315, *p* = .004, 95% CI -1.094, − 0.216). In addition, a better skin clearance (Delta BSA: Beta = − 0.303, *p* = .006, 95% CI − 0.515, − 0.091, Delta SAPASI: Beta = − 0.246, *p* = .043, 95% CI − 0.458, − 0.008), better quality of life (Delta DLQI: Beta = − 0.587, *p* < .001, − 0.767, − 0.407) and a reduction in anxiety/depression were associated with a better PBI (Delta HADS: Beta = − 0.363, *p* < .001, 95% CI − 0.571, − 0.156). Starting a new treatment episode with a biologic was also associated with a better PBI (Beta = 0.309, *p* = .005, 95% CI 0.196, 1.050) (Table 3).

In the group of patients with AD, most of the influencing variables were replicated. Better skin clearance (Delta BSA: Beta = − 0.386, *p* = .002, 95% CI − 0.628, − 0.144, Delta subjective SCORAD: Beta = − 0.459, *p* < .001, 95% CI − 0.669, − 0.206), better quality of life (Delta DLQI: Beta = − 0.583, *p* < .001, 95% CI − 0.794, − 0.371), and improved anxiety/depression (Delta HADS, Beta = − 0.353, *p* = .006, 95% CI − 0.605, − 0.105) were associated with a better patient benefit. However, gender and type of therapy had no significant effect on the PBI in AD (Table S3 in the supplementary material).

**Table 3:** Influencing variables of the Patient Benefit Index (PBI) in patients with psoriasis (PSO) (*n* = 82), using univariate regression analyses.

	Beta	T	*P*	95% CI
Age	− 0.091	− 0.814	0.418	− 0.312, 0.131
				F(1,81) = 0.662, *p* = .418 Corr. R^2^ = − 0.004
Sex	− 0.315	-2.970	0.004**	-1.094, − 0.216
				F(1,81) = 8.819, *p* = .004 Corr. R^2^ = 0.088
Delta BSA	− 0.303	-2.840	0.006**	− 0.515, − 0.091
				F(1,81) = 8.067, *p* = .006 Corr. R^2^ = 0.08
Delta SAPASI	− 0.246	-2.064	0.043*	− 0.458, − 0.008
				F(1,67) = 4.260, *p* = .043 Corr. R^2^ = 0.046
Delta DLQI	− 0.587	-6.486	< 0.001***	− 0.767, − 0.407
				F(1,81) = 42.066, *p* < .001 Corr. R^2^ = 0.336
Delta HADS	− 0.363	-3.490	< 0.001***	− 0.571, − 0.156
				F(1,81) = 12.180 *p* < .001 Corr. R^2^ = 0.121
Type of therapy (phototherapy/conventional systemic vs. biologics)	0.309	2.902	0.005**	0.196, 1.050
				F(1,81) = 8.421, *p* = .005 Corr. R^2^ = 0.084
Number of prior dermatologic treatments	0.026	0.228	0.820	− 0.197, 0.248
				F(1,81) = 0.052, *p* = .820 Corr. R^2^ = − 0.012

*p ≤ .05, ** *p* ≤ .01, ***p ≤ .001.

### Multiple linear regression model for PBI in PSO

In patients with PSO, in a stepwise multiple regression analysis, including sex, type of therapy, Delta BSA and Delta DLQI, 40.9% of the variance in the PBI could be explained by improvement in health-related quality of life (Delta DLQI) and initiation of biologic therapy (F (3,81) = 19.683, *p* < .001) (see Table S4 in the supplementary material). An alternative model, including Delta HADS, instead of Delta DLQI, explained only 29.3% of the variance (F(4, 81) = 9.411, *p* < .001) (Table S5 in the supplementary material).

### Mediator analyses

#### Reduction in anxiety/depression (Delta HADS) as mediator

**Fig. 1:**
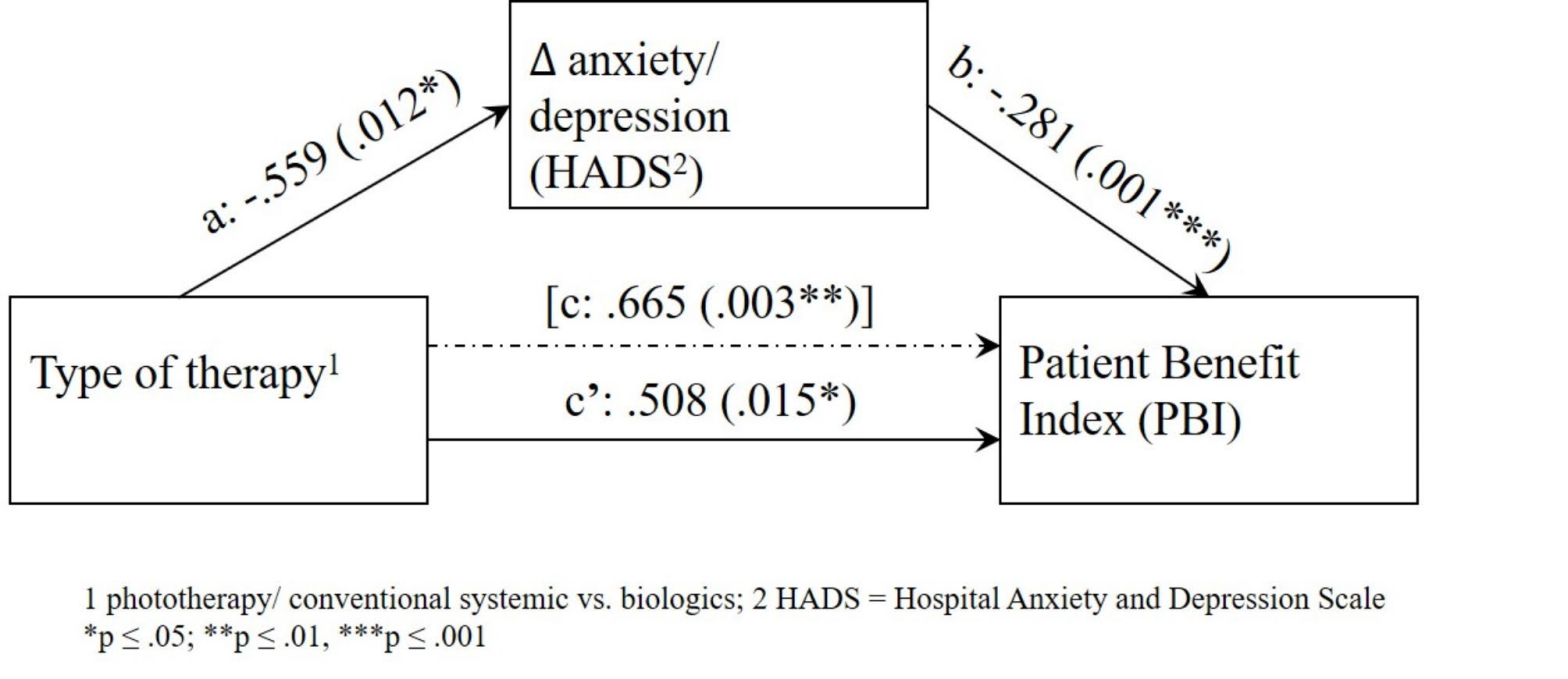
The effect of treatment type on the Patient Benefit Index (PBI), partially mediated by improvement in anxiety/depression [measured using the Hospital Anxiety and Depression Scale (HADS), before (T1) and after starting a new treatment episode] in patients with psoriasis (PSO).

A general total effect (path c) of type of therapy on the PBI could be demonstrated (p = .003, 95% CI .240, 1.090). In addition, the type of therapy had a significant effect on the reduction of anxiety/depression (Delta HADS) (p = .012, 95% CI − .991, − .128) (path a). The latter had a significant effect on the PBI (p = .001, 95% CI − .447, − .114) (path b). After entering the mediator Delta HADS in the model (c’), the total effect decreased, indicating a partial mediator effect (B = 0.508, SE = 0.205, t = 2.476, *p* = .015, 95% CI 0.100, 0.916). The indirect effect of the type of therapy on the PBI, mediated by the reduction in psychopathology (Delta HADS) (ab = 0.159, 95% 0.027, 0.335), turned out to be significant and proves the mediation (see Fig. 1, Table S6 in the supplementary material).

### Improvement in coping (Delta MSQ) as mediator

We found a significant direct effect of the improvement in BSA (Delta BSA) on the PBI (path c) (B = − 0.302, SE = 0.088, t = -3.4129, *p* = .001, 95% CI − 0.478, − 0.126). In addition, there was a significant relationship between the reduction in BSA (Delta BSA) and the MSQ subscales (*p* ≤ .010) (path a), except for the subscale ‘information seeking’ (*p* = .151). The largest effect of Delta BSA on the MSQ subscales was found for the subscale ‘quality of life’ (B = 0.365, SE = 0.071, t = 5.151, *p* < .001, 95% CI 0.224, 0.506). A higher improvement on the MSQ subscales was associated with a higher perceived benefit of therapy (PBI) (path b), which was not observed for the ‘pruritus’ and ‘information seeking’ subscales. The largest effect was found for ‘social anxiety’ (B = − .583, SE = .097, t = -6.029, p < .001, 95% CI − .776, − .390). Inclusion of the MSQ subscales in the model (path c’) led to a reduction in the total effect (path c), which was highest for ‘social anxiety’ (B = − 0.106, SE = 0.069, t = -1.529, *p* = .131, 95% CI − 0.243, 0.032) and ‘pruritis’ (B = − .147, SE = .097, t = -1.528, p = .131, 95% CI − .340, .045), suggesting full mediation. Finally, significant indirect effects of the impact of improved skin (Delta BSA) on the PBI, mediated by the coping facets, could be demonstrated, except for the subscale ‘information seeking’ (Table 4).

**Table 4:** The mediating effect of coping (Delta Subscale of the Marburg skin Questionnaire/MSQ) on the relationship between Delta BSA (body surface area) and perceived Patient Benefit Index (PBI), in patients with psoriasis (PSO) (*n* = 82).

Mediators	Total effect of x on y (c) (*p*, 95% CI)	Effect of X on M (a) (*p*, 95% CI)	Effect of M on Y (b) (*p*, 95% CI)	Direct effect (c’) (*p*, 95% CI)	Indirect effect^1^ (*p*, 95% CI)
Social anxiety	− 0.302 (0.001***, − 0.478, − 0.126)	0.337 (0.001***, 0.138, 0.535)	− 0.583 (< 0.001***, − 0.776, − 0.390)	− 0.106 (0.131, − 0.243, 0.032)	− 0.198 (-0.324, − 0.076)
Itch-scratch cycle	− 0.302 (0.001***, − 0.478, − 0.126)	0.317 (< 0.001***, 0.156, 0.479)	− 0.487 (0.057, − 0.988, 0.015)	− 0.147 (0.131, − 0.340, 0.045)	− 0.156, (-0.314, − 0.063)
Helplessness	− 0.299 (0.001***, − 0.474, − 0.125)	0.292 (0.010*, 0.073, 0.512)	− 0.369 (0.002**, − 0.593, − 0.144)	− 0.191 (0.023*, − 0.355, − 0.028)	− 0.109 (-0.229, − 0.021)
Anxiety/depression	− 0.302 (0.001***, − 0.478, − 0.126)	0.256 (0.005**, 0.080, 0.432)	− 0.367 (< 0.001***, − 0.547, − 0.187)	− 0.208 (0.021*, − 0.384, − 0.032)	− 0.095 (-0.183, − 0.020)
Quality of life	− 0.302 (0.001***, − 0.478, − 0.126)	0.365 (< 0.001***, 0.224, 0.506)	− 0.318 (0.001***, − 0.495, − 0.142)	− 0.186 (0.053, − 0.373, 0.002)	− 0.117 (-0.209, − 0.050)
Information seeking	− 0.302 (0.001***, -478, − 0.126)	0.168 (0.151, − 0.062, 0.398)	− 0.222 (0.091, − 0.479, 0.036)	− 0.265 (0.006**, − 0.449, − 0.080)	− 0.038 (-0.116, 0.012)

^1^Indirect effect is significant when the 95% CI does not contain zero.

*p ≤ .05, ** *p* ≤ .01, ***p ≤ .001.

## Discussion

The Patient Benefit Index (PBI) can be seen as a goal-directed outcome questionnaire that overcomes the shortness of the DLQI and provides a more accurate measure of patient-reported treatment success and effectiveness in PSO and other dermatological conditions. As a result, dermatological treatment can be tailored to the needs of the patient, leading to improved adherence. However, there is a lack of evidence regarding the influencing and modulating variables as well as the convergent validity of the PBI^41,42^. In the present study, we wanted to know whether the perceived patient benefit differed between patients with PSO and patients with AD, regardless of the therapy they were receiving. We were also interested in which patient needs were considered most important. Secondly, we investigated which influencing variables had a significant impact on patient benefit as measured by the PBI. In particular, we were interested in which variables, such as improvement in skin severity, type of dermatological treatment received, reduction in anxiety/depression, and improvement in health-related quality of life and skin-related coping, had the greatest effect. Thirdly and finally, we investigated whether there were mediating effects of anxiety/depression and coping on perceived patient benefit.

### PBI and its determinants in PSO and AD

Patients with PSO showed a mean PBI of 2.3 (range 0.0–4.0) and ~ 84% of the patients (independent of dermatological treatment) expressed a minimal patient-relevant benefit (PBI ≥ 1) from treatment. Among patients receiving systemtic therapy, approximately 76% reported a minimum PBI. This was lower than in a retrospective study of chronic plaque PSO treated with apremilast for six months (global PBI ≥ 1: 90.9%, mean PBI score 2.8)^21^. However, our findings fit with the mean PBI score of 2.5 and a rate of 86.7% of patients with psoriasis vulgaris, from a large cross-sectional, epidemiological study in 133 German dermatology practices and hospitals, reporting more than minimal benefit (PBI > 1)^7^. There was a slightly lower PBI in AD, but this was not statistically significant. This difference may reflect the time of recruitment, when only one potential biologic agent (dupilumab) was available for patients with AD, whereas patients with PSO had more options for effective dermatological treatments (S3-guideline PSO, AD) ^9 S3−Leitlinie „Atopische Dermatitis“ (AWMF−Registernr. 013–027) (2023) available under^: https://register.awmf.org/de/leitlinien/detail/013–027. Over time, systemic therapies have been gradually replaced by biologics, which provide faster and better clearance of psoriatic lesions and eczema through monoclonal antibodies and interleukin inhibitors^18^. As most patients with PSO consider the elimination of the skin lesion to be the most important treatment goal (‘to get better skin quickly’: 84.1%, ‘to be cured of all skin defects’: 81.7%), the PBI is higher for patients receiving biologics compared to other dermatological treatments. However, this could only be shown for the PSO group in the present study, not for the AD group. In line with this, Radtke et al.^7^ also showed a higher PBI in patients with PSO under biological therapy than under other treatments such as conventional systemic therapy or intensified local therapy.

In our present study, most patients with PSO considered ‘to get better skin quickly’ and ‘to be cured of all skin defects’ to be the most important treatment goals, whereas in a cross-sectional survey of self-reported PSO, ‘to have confidence in the therapy’ (86.3%) and ‘to regain control of the disease’ (85.9%) were considered most important^18^. In our present study, the latter were on place five and four of the top five treatment goals. The differences may be explained by the older mean age in our cohort of patients with PSO.

Women had lower levels of PBI than men. This is in contrast to a nationwide cross-sectional survey, which found no gender difference in PBI^13^. However, an earlier study found that women had higher expectations of therapy than men, regardless of treatment goals^18^. The higher treatment expectations may be associated with lower satisfaction and thus lower PBI scores. In our present study, men rated ‘to be cured of all skin defects’ (item 4 of the PBI) as more important than women, while the latter emphasised ‘to be able to sleep better’ (item 5) as more important (results not shown). Therefore, treatment options should therefore be carefully selected, taking into account gender-specific treatment needs and goals.

Regarding the effect of previous therapies, the present study did not show a significant effect. This contradicts the findings of Klein et al.^21^ who found a better PBI when patients had no prior treatment or only phototherapy. However, it can be assumed that in our present study an effect was masked due to imprecise documentation of the type of previous antipsoriatic treatment and no differentiation in the medical history with regard to conventional systemic treatment vs. biologic treatment vs. phototherapy. In our present PSO sample, almost three quarters (73.2%) had received prior antipsoriatic treatment. Thus, a lack of effect on the PBI may be due to unequal group size. Descriptively, the results even suggest a higher PBI in patients with prior treatment compared to those without prior treatment (*p* = .124).

Greater improvements in anxiety/depression and, most importantly, DLQI, were associated with greater perceived benefit from dermatological treatment. This is in line with previous findings, demonstrating a positive association between PBI and health-related quality of life^22,43^. Furthermore, our findings of a high association between the DLQI and the PBI suggest, that the PBI can be used to complement the assessment of the DLQI by taking into account treatment goals. Addressing a patient´s needs for the dermatological therapy is important, in order to improve patient-provider communication, align treatment goals and improve adherence. Considering individual treatment goals can help improve treatment decisions and create patient-centered treatment plans. Therefore, systemic and biologic agents should be selected according to their effect on the patient´s prioritised treatment goals (e.g. rapid improvement of skin lesions, improvement of depressed mood, sleep, sexuality). Future research should continue to evaluate the effects of dermatologic treatment not only on the improvement of skin lesions, but also on patient-oriented outcome measures^44,45^.

No findings have been published on the impact of anxiety/depression on the PBI. In the present study, the results even suggest a mediating effect of anxiety/depression on the PBI, which remained stable even after controlling for improvement in BSA. Thus, antipsoriatic treatment should also aim to reduce psychopathology, in order to optimise perceived treatment success. Evidence suggests that biologics reduce the risk of depressive symptoms, compared with conventional therapy^46,47^, and are associated with improvements in anxiety/depressive symptoms^48,49^. However, the incidence rates of depression adverse events are controversial when comparing different treatments^46,50,51^. Another aspect concerns findings that report a similar underlying pathophysiological mechanism between PSO and depression. Both are associated with increased systemic inflammation^52,53^. Above, there may be evidence of an effect of of circulating pro-inflammatory cytokines on 5-HTT availability and depressiveness^54^.

Finally, improvement in DLQI appears to have the greatest impact on perceived treatment benefit and is more important than PSO/AD severity alone, followed by the type of dermatological treatment. This contradicts the finding that most patients (> 81%) consider complete and rapid clearance of psoriatic plaques to be the most important treatment goal. In another model, looking at changes in anxiety/depression instead of quality of life, a similar positive effect of improved psychopathology and skin clearance on the PBI was shown. Above, we even found a mediating effect (with the highest indirect effect) of the quality of life (as measured with the Marburg Skin Questionnaire) on the PBI. Thus, as concluded by Andersch-Björkman et al.^49^, our findings also emphasise the importance of addressing depressive and anxiety symptoms, together with quality of life and psoriasis severity as outcome measures, in the treatment of PSO.

In order to improve anxiety/depression and quality of life, patients should be supported in coping appropriately with the skin condition, e.g. by informing the social environment about the skin disease, being sensitive about diet, actively caring for the skin, showing less social withdrawal or passive coping, using normalisation/optimistic evaluation of the skin condition, etc^55,56^. In an older study, patients with PSO who used normalisation/optimistic coping more often reported higher levels of mental health^57^. In another study, patients with PSO were less likely to use active coping strategies, planning, positive reinterpretation and humour than healthy controls^56^. Inadequate coping strategies, such as pathological/high worry, were associated with poorer treatment outcomes in a previous study^58^. Patients with PSO, as with other chronic inflammatory skin diseases, should be well educated and trained to use appropriate problem and emotion-focused strategies to better live with and manage the disease in their daily lives. For example, complementary psychological interventions and educational techniques should aim to proactively manage social discrimination and stigma, help reassess chronic health problems and reduce social withdrawal.

### Strength and limitations

Finally, our results should be evaluated in the context of the methodological strengths and limitations. The present study cohorts were assessed prospectively, using highly reliable self-report instruments and dermatologist assessments of disease. Patients were enrolled consecutively in both inpatient and outpatient settings. Two dermatological sub-samples were used to facilitate the generalisability of the study results. For the first time, influencing and mediating variables on the PBI were addressed. However, conclusions should be drawn with caution given the small sample size and the high drop-out rate (35.9%). Another limitation is that objective assessment of skin disease severity was not consistently available. PASI scores could be obtained in only 34.1% of the PSO patients and objective SCORAD scores in only about 70.5% of the AD patients. Consequently, the subjective severity score BSA was used as the primary outcome. This approach can be justified by the fact that in the present study the BSA showed a moderate correlation with the objective severity score in the cohort of PSO patients (Spearman’s rho = 0.267, *p* = .001). We therefore decided to use the BSA, which is consistent with previous studies^59^. We enrolled a rather heterogeneous convenience sample, i.e. selection bias cannot be completely ruled out. In addition, the generalisability of the present results is rather limited to the time frame and the therapies approved during the study period. At that time, just over four years ago, the only modern systemic therapy for AD was the first biologic, dupilumab. Other biologics, as well as JAK inhibitors, were still being tested in pivotal trials. Similarly, in PSO, therapies such as tildrakizumab, guselkumab and risankizumab were only approved during the study period and were not prescribed in our study sample. Bimekizumab and Deucravacitinib would not be approved until after 2019 ^9^. Newer biologics are more effective and target pathways more specifically than older ones. Nevertheless, it can be assumed that the higher skin clearance rate may be associated with higher Patient Benefit Indices, higher skin-related quality of life and lower anxiety/depression^48,60^. Following, study results may not be comparable if patients with newer biologics and JAK inhibitors had been included. Finally, future studies should replicate the present findings by including multiple dermatological study centres, enrolling sufficiently sampled PSO/AD cohorts, using objective measures of PSO/AD severity, and taking into account prior dermatological treatments in more detail. In addition, the generalisability of the study results should be verified, including sufficiently large subsamples of patients receiving conventional or modern systemic treatments, e.g. biologics and Jak inhibitors.

## Conclusion

Finally, it is concluded that the PBI is an adequate, goal-oriented measure of treatment success in patients with PSO and AD, which complements the DLQI by taking into account patients’ individual treatment expectations and needs. Dermatological treatment should take into account gender-specific treatment goals.These should include not only improvement of skin lesions but also improvement of anxiety/depression and quality of life. For this purpose, complementary psychological/psychosomatic interventions should be offered, especially to improve adequate coping with chronic inflammatory skin diseases.

## Electronic supplementary material

Below is the link to the electronic supplementary material.

Supplementary Material 1

## Data Availability

The data are available on request. The corresponding author GBW should be contacted in order to obtain the data from this study.
