# Supplementary material for: Determinants of perceived patient benefit in a longitudinal cohort study of patients with psoriasis and atopic dermatitis
Source: Sci Rep. 2025 Jan 10;15:1553. doi: 10.1038/s41598-024-84794-2 (PMC11717929; doi:10.1038/s41598-024-84794-2)
Supplement: Supplementary file 1 — Supplementary Material 1 [file 41598_2024_84794_MOESM1_ESM.docx]

**Supplementary Material**

**Table S1-S4**

**Table S1:** Clinical characteristics of patients with psoriasis (PSO) (n = 82) and atopic dermatitis (AD) (n = 61) before (T1) and about 16 weeks after (T2) beginning of a new treatment episode.

| **Patients´ characteristics** | **Patients with PSO (n = 82)** | **Patients with AD (n = 61)** | **U/Z/T/ ꭓ² (p)** |
| --- | --- | --- | --- |
| Age of onset of PSO (yrs), mean (SD), min-max range | 31.4 (15.3), 2.7-66.1 | -- | -- |
| Duration of PSO (yrs), median (IQR), min-max range | 17.6 (7.0-26.9), .14-60.9 | -- | -- |
| Body Surface area at T1/ at T2, median (IQR),  number of cases with BSA ≥ 3% at T1/ T2 | 18.5 (9.0-43.0)/  5.0 (2.0-11.0)  75 (91.5)/ 56 (68.3) | 28.0 (13.0-54.8)^1^/ 13.0 (5.5-26.5)  59 (96.7)^1^/ 54 (88.5) | T1: P vs. AD  2076.500, (.113) (U)  T2: P vs. AD  1477.000,  **<.001***** (U)  P: -6.610 (**<.001*****) (Z)  AD: -4.149 (**<.001*****) (Z) |
| PASI/ objective SCORAD at T1/at T2, median (IQR),  number of cases (%) with PASI ≥ 10/  objective SCORAD ≥ 25 at T1/ at T2 | 14.5 (10.8-20.9)^1^/ 4.0 (1.4-9.8)^2^  61 (74.4)^1^/ 7 (8.5)^2^ | 60.4 (47.1-69.2)  58.8 (mean), 17.8 (SD)^2^/ 42.7 (mean), 13.4 (SD), 43.3 (30.6-50.6)^3^  41 (67.2)^6^/ 16 (26.2)^7^ | P: -3.714 (**<.001*****) (Z)  AD: -1.804 (.071) (Z) |
| Subjective PASI/ SCORAD at T1/ T2, median (IQR),  number of cases with subjective PASI > 3 at T1/ at T2 | 16.4 (7.4-26.0)^3^/ 2.6 (.8-6.6)^4^  78 (95.1)^3^/ 33 (40.2)^4^ | 60.2 (mean), 17.7 (SD)^4^/ 41.4 (mean), 21.8 (SD)^5^ | P: Z = -6.046 (**< .001*****)  AD: T = 5.246 **(< .001***)** |
| DLQI at T1/ at T2, median (IQR) | 7.5 (3.8-13.0)/ 2.0 (.0-5.0) | 11.0 (6.5-17.5), 12.7 (mean), 6.9 (SD)/ 5.0 (2.0-9.0) | T1: 1756.500 (**.002****) (U)  T2: 1551.500 (**<.001*****) (U)  P: -5.856 (**< .001*****) (Z)  AD: -5.470, (**<.001*****) (Z) |
| HADS at T1/ at T2, median (IQR) | 10.0 (5.0-15.0)/ 10.0 (5.0-14.0), 10.3 (mean), 7.2 (SD) | 13.0 (8.0-19.0), 13.2 (mean), 6.6 (SD)^6^/ 9.0 (5.0-17.0) | T1: 1861.500 (**.020***)  T2: 2268.000 (.341)  P: -.386 (.699) (Z)  AD: -2.292 (**.022***) (Z) |
| Somatic comorbidities, n (%)  Psoriasis et arthritis, yes  Allergies, yes  Autoimmune, yes  Infectious disease, yes  Thyreoid disease, yes  Cardiovascular disease, yes  Circulatory disease, yes  Lung disease, yes  Liver disease, yes  Kidney disease, yes  Digestive disease, yes  Metabolic disease, yes  Musculoskeletal disease, yes  Blood disease | 20 (24.4)  23 (28.0)^5^  12 (14.6)^6^  4 (4.9)  11 (13.4)  13 (15.9)  37 (45.1)  9 (11.0)^7^  17 (20.7)  4 (4.9)  3 (3.7)  16 (19.5)^8^  7 (8.5)  5 (6.1) | --  52 (85.2)  48 (78.7)  4 (6.6)  10 (16.4)  4 (6.6)^7^  13 (21.3)^8^  24 (39.3)^9^  4 (6.6)  3 (4.9)  2 (3.3)  5 (8.2)  5 (8.2)^10^  4 (6.6) | 46.031 (**<.001*****) (ꭓ²)  59.137 (**<.001*****) (ꭓ²)  .187 (.724) (Fisher´s exact test)  .248 (.619) (ꭓ²)  4.122 (.127) (ꭓ²)  9.689 (**.008****) (ꭓ²)  16.081 (**<.001*****) (ꭓ²)  5.609 (**.018***) (Fisher´s exact test)  .000 (1.000) (Fisher´s exact test)  .015 (1.000) (Fisher´s exact test)  3.688 (.060) (Fisher´s exact test)  1.356 (.508) (ꭓ²)  .013 (1.000) (Fisher´s exact test) |

*Psoriasis*, missing values: ^1^ n = 6, ^2^ n = 54, ^3^ n = 2, ^4^ n = 12, ^5^ n = 2, ^6^ n = 2, ^7^ n = 1, ^8^ n = 1; *Atopic dermatitis*, missing values: ^1^ n = 1, ^2^ n = 18, ^3^ n = 44, ^4^ n = 1, ^5^ n = 4, ^6^ n = 2, ^7^ n = 1, ^8^ n = 1, ^9^ n = 1, ^10^ n = 1; *p ≤ .05, ** p ≤ .01, ***p ≤ .001

**Table S2:** Sociodemographic and life-style characteristics of patients with psoriasis (PSO) (n = 82) and dropped out patients with PSO (n = 46) before (T1) the beginning of a new treatment episode.

| **Patients´ characteristics** | **Patients with PSO (n = 82)** | **Drop outs (PSO) (n = 46)** | **U/Z/T/ ꭓ² (p)** |
| --- | --- | --- | --- |
| Age, median (IQR), min-max Range | 54.6 (38.2-62.6), 22.9-74.9 | 50.2 (34.2-57.0), 20.2-71.3 | 1596.000 (U) (.150) |
| Gender, n (%)  Women  Men | 29 (35.4)  53 (64.6) | 19 (41.3)  27 (58.7) | .443 (.505) (ꭓ²) |
| Education, n (%)  No school degree/ Less than 10 years  10 years  High school diploma | 12 (14.6)  51 (62.2)  19 (23.2) | 6 (13.0)  20 (43.5)  14 (30.4)^1^ | 2.080 (.353) (ꭓ²) |
| Partnership, n (%)  Married  Cohabited  No partnership | 36 (43.9)  31 (37.8)  15 (18.3) | 20 (43.5)  13(28.3)  12 (26.1)^2^ | 1.627 (.443) (ꭓ²) |
| Regular sport, n (%)  Yes  No | 23 (28.0)  59 (72.0) | 12 (26.1)  32 (69.6)^3^ | .009 (.926) (ꭓ²) |
| Regular alcohol, n (%)  Yes  No | 39 (47.6)  43 (52.4) | 24 (52.2)  21 (45.7)^2^ | .387 (.534) (ꭓ²) |
| Smoking, n (%)  Yes  No | 21 (25.6)  57 (69.5)^1^ | 20 (43.5)  23 (50.0)^4^ | 4.748 (**.029***) (ꭓ²) |
| BMI kg/ m²,  median (IQR), min-max Range | 27.1 (24.6-31.6), 17.0-53.9 | 29.1 (6.8), 20.1-52.5  29.0 (24.8-31.8)^4^ | 1668.000 (.621) (U) |
| Body Surface area at T1, median (IQR) | 18.5 (9.0-43.0) | 24.0 (9.8-50.0) | 1724.000 (.421) (U) |
| Objective PASI at T1, median (IQR) | 14.5 (10.8-20.9)^2^ | 14.2 (8.2-21.1)^4^ | 1631.500 (.989) (U) |
| DLQI at T1, median (IQR) | 7.5 (3.8-13.0) | 14.0 (7.0-16.5)^2^ | 1293.000 (**.005****) |
| HADS at T1, median (IQR) | 10.0 (5.0-15.0) | 14.0 (9.5-16.0)^2^ | 1321.000 (**.008****) |

*Psoriasis*, missing values: ^1^ n = 4, ^2^ n = 6; *Drop outs*, missing values ^1^ n = 6, ^2^ n = 1, ^3^ n = 2, ^4^ n = 3

**Table S3:** Influencing variables of the Patient Benefit Index (PBI) in patients with atopic dermatitis (AD) (n = 61), using univariate regression analyses.

|  | **Beta** | **T** | **P** | **95% CI** |
| --- | --- | --- | --- | --- |
| Age | .202 | 1.586 | .118 | -.053, .457 |
|  |  |  |  | F(1,60) = 2.514, p = .118  Corr. R² = .025 |
| sex | .084 | .649 | .519 | -.350, .687 |
|  |  |  |  | F(1,60) = .422, p = .519  Corr. R² = -.010 |
| Delta BSA | -.386 | -3.191 | **.002**** | -.628, -.144 |
|  |  |  |  | F(1,59) = 10.182, p = .002  Corr. R² = .135 |
| Delta objective SCORAD | -.459 | -3.793 | **<.001***** | -.669, -.206 |
|  |  |  |  | F(1,55) = 14.384, p < .001  Corr. R² = .196 |
| Delta DLQI | -.583 | -5.507 | **<.001***** | -.794, -.371 |
|  |  |  |  | F(1,60) = 30.327 p < .001  Corr. R² = .328 |
| Delta HADS | -.353 | -2.845 | **.006**** | -.605, -.105 |
|  |  |  |  | F(1,58) = 8.094, p = .006  Corr. R² = .109 |
| Type of therapy (phototherapy/ local therapy vs. systemic/ biologics) | .141 | 1.092 | .279 | -.255, .867 |
|  |  |  |  | F(1,60) = 1.192, p = .279, Corr. R² = .003 |

BSA = Body Surface Area, DLQI = Dermatology Life Quality Index, HADS = Hospital Anxiety and Depression Scale, PBI = Patient Benefit Index, SCORAD = Scoring Atopic Dermatitis; ** p ≤ .01, ***p ≤ .001

**Table S4:** Multiple stepwise regression analysis including influencing variables of the Patient Benefit Index (PBI) in patients with Psoriasis (PSO) (n = 82).

|  | **Beta** | **T** | **P** | **95% CI** |
| --- | --- | --- | --- | --- |
| Delta DLQI | -.497 | -5.559 | **<.001***** | -.675, -.319 |
| Type of therapy (phototherapy/ conventional systemic vs. biologics) | .225 | 2.571 | **.012*** | .102, .805 |
| sex | -.222 | -2.524 | **.014*** | -.824, -.097 |
| Corr. R² = .409, F (3,81) = 19.683, p < .001***, stepwise | | | | |

DLQI = Dermatology Life Quality Index, *p ≤ .05, ***p ≤ .001

**Table S5:** Multiple stepwise regression analysis including influencing variables (Delta HADS instead of Delta DLQI) of the Patient Benefit Index (PBI) in patients with Psoriasis (PSO) (n = 82).

|  | **Beta** | **T** | **P** | **95% CI** |
| --- | --- | --- | --- | --- |
| Delta HADS | -.234 | -2.379 | **.020*** | -.430, -.038 |
| Type of therapy (phototherapy/ conventional systemic vs. biologics) | .245 | 2.517 | **.014*** | .103, .885 |
| sex | -.302 | -3.215 | **.002**** | -1.018, -.239 |
| Delta BSA | -.239 | -2.525 | **.014*** | -.428, -.051 |
| Corr. R² = .293, F (4,81) = 9.411, p < .001***, stepwise | | | | |

BSA = Body Surface Area, HADS = Hospital Anxiety and Depression Scale, PBI = Patient Benefit Index, *p ≤ .05, ** p ≤ .01

**Table S6:** Mediating effect of Delta HADS on the relationship between type of therapy and perceived Patient Benefit Index (PBI), in patients with psoriasis (PSO) (n = 82).

| **Mediator** | **Total effect of x on y (c)**  **(p, 95% CI)** | **Effect of X on M (a)**  **(p, 95% CI)** | **Effect of M on Y (b)**  **(p, 95% CI)** | **Direct effect (c’)**  **(p, 95% CI)** | **Indirect effect (ab)^1^**  **(p, 95% CI)** |
| --- | --- | --- | --- | --- | --- |
| **Delta HADS** | .665 (.003**, .240, 1.090) | -.559 (.012*, -.991, -.128) | -.281 (.001***, -.447, -.114) | .508 (.015*, .100, .916) | .159 (.027, .335) |

HADS = Hospital Anxiety and Depression Scale, PBI = Patient Benefit Index, ^1^ Indirect effect is significant when the 95% CI does not contain zero. *p ≤ .05, ** p ≤ .01, ***p ≤ .001
